# P-240. Comparing Infection Rates Against Mechanical Complications By Site of Placement of Central Venous Catheters in the ICU, a Moot Point Based on Excessive Mortality?

**DOI:** 10.1093/ofid/ofae631.444

**Published:** 2025-01-29

**Authors:** Michael T Stevens, Rafael Ponce, Arjun Patel, Patrick Kinsella

**Affiliations:** Mercer University School of Medicine, Macon, Georgia; Mercer University School of Medicine, Macon, Georgia; Mercer University School of Medicine, Macon, Georgia; Mercer University School of Medicine, Macon, Georgia

## Abstract

**Background:**

In the ICU the decision regarding site selection for central venous catheters (CVC) is largely influenced by the historic infection rates by site. It is thought that femoral placement has the highest incidence of line infections and therefore is often avoided. However, few studies have aimed to look at the overall risk to the patient regarding site placement taking not only risk of infection, but also risk of mechanical injury and complications into consideration.

**Methods:**

We did a retrospective review of all patients who had a new central line (either triple lumen catheter or hemodialysis catheter) placed in the ICU for any indication between 1/31/2022 - 5/4/2023. Key information including demographics, Charlson Comorbidity index, type of catheter, site, date of insertion, and date of removal was obtained. Manual chart review was done for each patient in order to ascertain presence of CLABSI as well as mechanical complications.

**Results:**

472 lines were placed during the study period. The average age was 63.5 years. The average Charlson Comorbidity index was 5. There was no statistical significant difference in infection rates among femoral 5/196 (2.5%) and internal jugular (IJ) 9/247 (3.6%) CVCs, although IJ lines had a trend towards higher rates of mechanical complications 21/247 (8.5%) vs 11/195 (5.6%), respectively. Rate of infection was twice as high, 6/267 (2.3%) vs 9/205 (4.4%) in the BMI >30 group vs those with a BMI < 30. The 6-month all-cause mortality for patients requiring a central line in the ICU was >80%, regardless of the site of placement, presence of line infection, or mechanical complication.

**Conclusion:**

Infection rates between IJ and femoral lines were comparable. Femoral lines had numerically less mechanical complications as compared to IJ lines. BMI > 30 had a trend towards an increased risk of CLABSI. Overall mortality was > 80% for patients who required a CVC for any reason in the ICU setting. Clinicians should take a balanced approach regarding infection and mechanical complication risk when placing a central line. Further research into determining infection rates for patients with higher BMI is needed. Intensivists should consider early palliative care consultation for patients requiring a central line.

**Disclosures:**

**All Authors**: No reported disclosures

